# Does Shining Light on Gold Colloids Influence Aggregation?

**DOI:** 10.1038/srep05213

**Published:** 2014-06-09

**Authors:** Susmita Bhattacharya, Suda Narasimha, Anushree Roy, Soumitro Banerjee

**Affiliations:** 1 Department of Physics, Indian Institute of Technology Kharagpur, India; 2 Department of Physical Sciences, Indian Institute of Science Education and Research, Kolkata, Mohanpur Campus, India; 3 Department of Physical Sciences, Indian Institute of Science Education and Research, Kolkata, Mohanpur Campus, India, and King Abdulaziz University, Jeddah, Saudi Arabia

## Abstract

In this article we revisit the much-studied behavior of self-assembled aggregates of gold colloidal particles. In the literature, the electrostatic interactions, van der Waals interactions, and the change in free energy due to ligand-ligand or ligand-solvent interactions are mainly considered to be the dominating factors in determining the characteristics of the gold aggregates. However, our light scattering and imaging experiments clearly indicate a distinct effect of light in the growth structure of the gold colloidal particles. We attribute this to the effect of a non-uniform distribution of the electric field in aggregated gold colloids under the influence of light.

Self-similar fractal structure of aggregated gold colloids was first observed using transmission electron microscopy for selected sizes of the particles in the sol^1^. Later, the physical properties, e.g., fractal dimension (*D_f_*) and radius of gyration (*R_g_*) of colloidal aggregates in the sol were probed by static and dynamic light scattering, small angle x-ray scattering, and neutron scattering techniques^2^,^3^,^4^,^5^. Various models have been developed to explain the observed short-range and long range structures of the particle aggregates in a stable colloidal dispersion^6^,^7^,^8^,^9^,^10^,^11^,^12^. It is well-established that the effective forces acting between the particles at the microscopic scale determine the morphology of aggregates during pre-nucleation, nucleation and super-cluster formation in the solvent. In early reports, the short range structure of particle aggregation was assumed to be defined by the Derjaguin-Landau-Verwey-Overbeek (DLVO) potential between the particles^6^,^13^,^14^. For nanoparticles stabilized by long-chain ligand molecules, instead of the electrostatic repulsion term in DLVO potential, the free energy of mixing of ligand molecules was considered^8^,^9^. Detailed phenomenological models and computer simulation studies on the interaction potential between ligated gold particles in 3D was reported by Khan et al.^8^,^9^,^10^. In yet another approach, molecular dynamics (MD) simulation^15^ based on Ginzburg-Landau model—which is a dissipative stochastic model based on Langevin equation—was used to describe the process of nucleation^16^. In this approach, noise describes the effect of the microscopic degrees of freedom of the host fluid. It explained the triangular or honeycomb like packing of particles in 2D and BCC-like packing (icosahedral arrangement when BCC is inhibited) in 3D, upon nucleation^17^,^18^,^19^. Reaction Limited Aggregation (RLA) model describes the slower growth kinetics in the presence of such potential barriers^20^. Fast Diffusion Limited Aggregation (DLA) was used to define the dendritic growth of the colloidal aggregation. In the DLA model one considers random walk of the free particles due to Brownian motion, before they cluster together to form aggregates^2^,^21^,^22^,^23^.

In this article, we re-investigate the fractal structures formed by gold colloidal particles and experimentally obtain the fractal characteristics using static light scattering and microscopic imaging experiments. We show a distinct effect of light in determining the characteristics of the self assembled gold aggregates, which, to best of our knowledge, has not been discussed earlier in the literature.

## Results and Discussion

### Characteristics of freshly prepared gold sol

**Figure 1:**
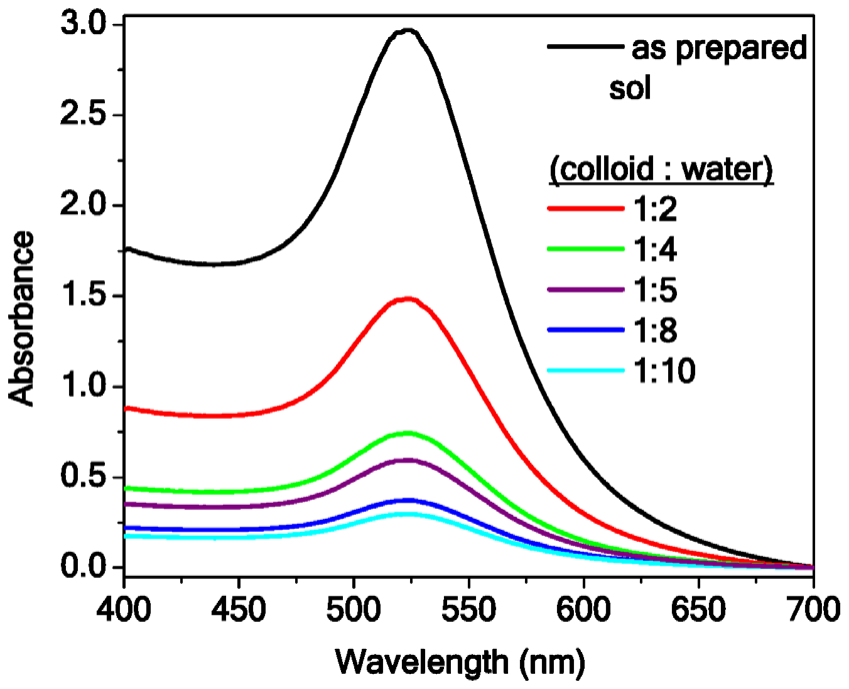
Surface plasmon resonance spectra of gold colloid of different concentrations.

Fig. 1 describes the surface plasmon resonance band of freshly prepared gold colloidal particles of various concentrations. All spectra exhibit the absorption peak at 525 nm. Neglecting the scattering of light by small particles and using Mie's solution to Maxwell's equations for dielectric spheres in a medium one can estimate the average size (diameter *d*) of the particles (monomers), using the observed resonance maxima^24^,^25^. The average size of the particles in all concentration of the sol is estimated to be 22 nm. The resonance energy depends on various factors, e.g., average size of the particles in the sol, surface charge on the particles, dielectric constant of the medium, etc. We observe in Fig. 1 that none of the parameters of the spectral line profile, except the intensity of the resonance peak, change with dilution. Thus, it is reasonable to believe that the dilution decreases the volume fraction of the particles in the sol, but the characteristics of the colloidal system governing the interactions between the colloidal particles, the ligand, and the medium remain unchanged.

### Effect of light on aggregation

**Figure 2:**
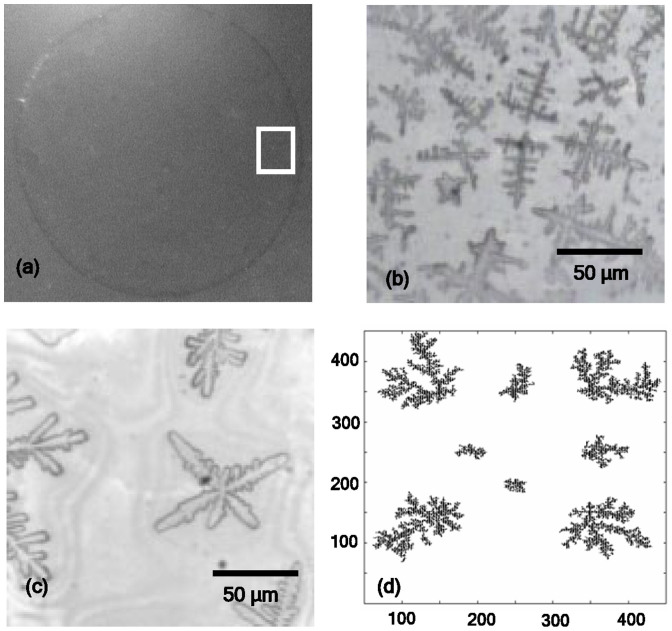
(a) Microscopic image of the colloidal droplet after evaporation of the solvent. The close-up optical microscopic views of the aggregates are recorded near the edge (white marked region) of the dried drop. Morphology of gold colloid fractals grown in (b) normal light and c) darkness. (d) DLA clusters as generated from simulation with sticking probability 0.1 with total length as 450 units and particle size as one unit.

We studied close-up views of gold colloidal aggregates. Drops of the above colloidal sol (diluted to 1:8 volume ratio) were transferred on a glass plate and were allowed to dry. Fig. 2(a) presents the characteristic image of the drop on the glass substrate after evaporation. The microscopic images are recorded at the edge of the dried drop [position shown as the white square in Fig. 2(a)]. The aggregates were grown both in light as well as in darkness. To grow the aggregate in darkness, the drop of the colloid was transferred on the plate just after the preparation and then was kept in dark approximately 10 hours till it fully dried, before exposing to light for imaging. In light the drops were fully dried within 4 hrs. Temperature and humidity of both sets were maintained same. Fig. 2(b) and (c) show characteristic optical microscopic images of the particle aggregates grown under the above conditions. It is clear from Fig. 2 that the morphology of the aggregate is different when grown in the presence and absence of light. In light, the general shape of the structure is characterized by perpendicular growths at different points of a main stem. On the other hand, in darkness, it is more like a snowflake-like growth around a nucleating area. We performed power spectral analysis of the microscopic images (details available in supplementary section, S1) to verify the length scale which defines the observed fractal features. The fractal dimension was estimated to be 1.7 ± 0.2 and 1.9 ± 0.2 for Fig. 2(b) and (c), respectively. The morphologies of the particle aggregates grown on NaOH treated glass plate and silicon substrate were observed to be very similar to that shown in Fig. 2, indicating that interactions between glass surface, the particles, and the medium do not play any major role in determining the observed morphology of the aggregates.

We investigated the pattern formation or directional solidification process through Monte-Carlo simulation of DLA growth^22^ assuming a sticking probability based on biased diffusion of the particles. The sticking probability was assumed to be governed by the short-range inter-particle interaction, and for our simulation we considered various values of it. The snapshot of a typical DLA cluster is shown in Fig. 2 (d), where agglomeration of 30000 particles with sticking probability 0.1 was considered. The snapshot mimics the observed fractal structures grown in dark (Fig. 2 (c)) quite well but does not show the very specific directionality in Fig. 2(b). The simulation, carried out for other values of sticking probability, also could not simulate the pattern in Fig. 2(b).

In the literature various factors are discussed to explain the difference in morphology of colloidal aggregates formed in an evaporating droplet. The drying of the solvent from the liquid drop on the glass slides starts from the edge. The liquid area slowly shrinks to the middle. Due to evaporation of the solvent, the concentration increases. Particles aggregate as the attractive capillary pressure in the channel between two colloidal particles exceed the repulsive forces discussed earlier^26^. These effects have been modeled using DLVO potential and capillary forces between particles in an evaporating colloidal system for silica and polystyrene colloidal system, to explain the dynamics of aggregation during evaporation at different drying fronts^26^. However, it does not explain the directional growth of the fractal structures in presence of light observed in our experiment. Order-disorder structural phase transition from the edge towards the centre of a ring-shaped colloidal stain (of coffee) on a glass plate has been related to the deposition rate of the colloidal particles^27^. The ordered hcp phase is reported to be formed by the particles at the edge of the stain when the deposition rate is slow, whereas the disordered phase is related to the aggregation inside the edge for faster deposition rate during the final stage of evaporation process. Deposition rate is related to the evaporation rate of the solvent. Our observation for colloidal gold particles is exactly opposite to what we expect from the above. For slower evaporation (10 hrs) in darkness we find more disordered structure (Fig. 2(c)) than for aggregates grown faster (Fig. 2(b)).

**Figure 3:**
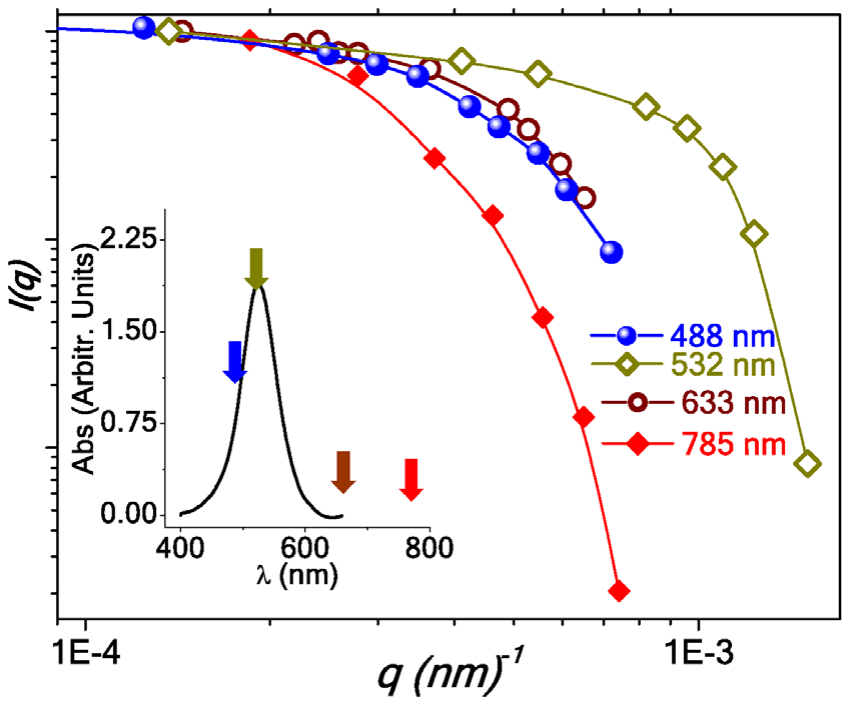
*I(q)* versus *q* plots for gold colloid recorded for different excitation wavelengths. Inset exhibits background subtracted plasmon resonance band of the gold colloid. The arrows indicate the excitation wavelengths used in scattering measurements.

To investigate whether light indeed has any effect on colloidal aggregation, we studied the characteristics of the fractal structures of the gold aggregates using light scattering measurements with different excitation wavelengths. Light scattering measurements are commonly used to study colloidal aggregation. It measures the scattering intensity *I(q)* from a sample, as a function of the scattering wavevector, *q* = (4*πn*/*λ*)sin(*θ*/2). Here λ is the incident wavelength, *n* is the refractive index of the solvent (water) and *θ* is the scattering angle. The scattering intensity *I(q)* is proportional to the scattering factor^3^ *S(q)*, where *S(q) ~ (qR_g_)^−Df^* for *qR_g_* ≫ 1. Fig 3 presents the *I(q)* versus *q* plot in a logarithmic scale for the sol of 1:8 dilution, recorded for different wavelengths (488, 532, 633 and 785 nm) using static light scattering measurements. All plots in Fig. 3 corresponding to different excitation wavelengths exhibit a power-law behavior at high *q*, and nearly *q*-independent isotropic behavior for low *q*. The range of *q* over which the cross-over between these two regimes occurs, is sensitive to the values of *R_g_* and *D_f_* (since *I(q)*∞*S(q)*). However, it is not expected to differ appreciably with excitation wavelength^30^. Interestingly, we observe the cross-over at much high *q* regime for 532 nm excitation wavelength, in comparison with the cross-over *q* values found with λ = 488 nm. To check the possibility of any photo-induced effect on ligand molecules, we have measured the UV-vis absorption of the ligand (sodium citrate) in solution over the spectral range between 200 and 900 nm. We do not find any absorption of radiation over the given range. This excludes any photo-induced effect on the ligand in the sol.

At this point we refer to plasmon resonance band (shown in Fig. 1) of gold particles in the sol. In the inset of Fig. 3 we plot the background-subtracted spectrum of gold colloid with dilution 1:8. The arrows mark the excitation wavelengths, used in the scattering measurements in Fig. 3. As discussed, the anomalous behavior of *I(q)* vs. *q* plot in Fig. 3 is observed for excitation wavelength 532 nm, which is at the peak of plasmon resonance band of the gold colloid. It is well-known that, due to the surface plasmon resonance, there is a non-uniform distribution of electric field intensity around the array of metallic nanoparticles under electromagnetic radiation^28^,^29^. For example, the intensity of the electric field at the space between two metallic colloidal nanoparticles is an order of magnitude higher than the field surrounding the rest of the particle boundary. These locations between particles with high field amplitude are known as ‘hot spots'. In presence of light, while approaching an existing cluster, an isolated particle is expected to experience the effect of this non-uniform electric field and will preferentially get attached at these hot spots, where the field is maximum. Such field-specific attachment may cause directional growth of the aggregation, which can affect the fractal morphology, as observed in Fig. 2 and the fractal characteristics, reflected in Fig. 3. Here we would like to point out that in a brief Comment^30^ on Ref. 2, Wilcoxon et al. indeed mentioned the effect of resonance wavelength in determining fractal characteristics based on light scattering measurements, though this fact was never discussed in detail.

**Figure 4:**
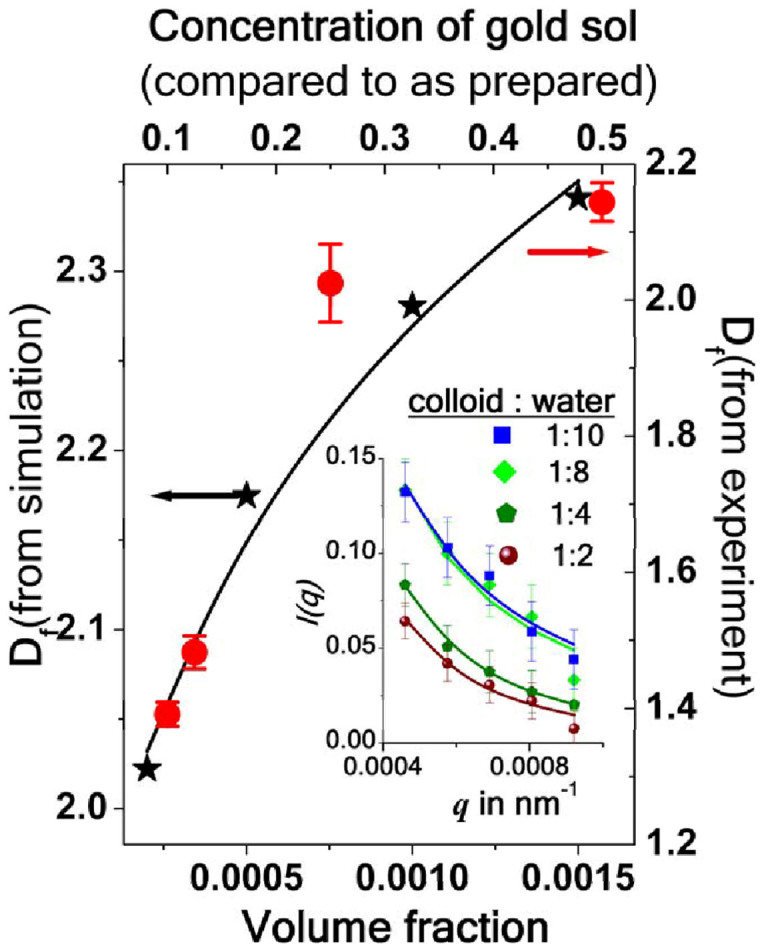
Variation of *D_f_* with concentration as obtained from light scattering measurements (red symbol). D_f_ as obtained from 3D simulation are shown by black symbols. The solid lines are the fit to the data points with the same exponent 0.36. Inset of the figure shows the measured *I(q)* vs *q* plots for different concentrations of the sol. Solid lines are fit to the data points using *I(q)* = *A(qR_g_)^−Df^*.

In several reports^2^ only DLVO type potentials are used to describe fractal characteristics obtained from scattering measurements. To investigate the extent up to which it is valid, we have carried out static light scattering measurements on gold sol of different concentrations. We record *I(q)* for different values of *q* for sols of different concentrations (inset of Fig. 4) using λ = 633 nm. We fit our data set for each concentration with the equation *I(q)* *=* *A(qR_g_)^−Df^*, (where *A* is a constant that depends on the average mass of the aggregates) keeping *A*, *R_g_* and *D_f_* as free fitting parameters. The variation of *D_f_* with concentration, thus obtained, is shown in Fig. 4 by filled circles. To estimate the fractal dimension of the aggregates of gold particles in the sol we considered DLVO type of interaction potential between particles, in a BD simulation, based on translational diffusion motion of the particles. Details of the simulation are available in the supplementary section (S2). The variation of *D_f_* with volume fraction, thus simulated, is shown by the black asterisks in Fig. 4. Both the simulated and experimental data points follow very similar behavior and could be fitted with the same power law. Thus, we conclude that the Brownian dynamics simulation including DLVO type of interaction potential between gold particles in the sol *qualitatively* explains the short-range dynamics of self-assembled aggregates. Based on our observation reported in Fig. 2 and Fig. 3, we are of the opinion that the effect of light needs to be considered in order to understand the detailed morphology and characteristics of such self-assembled gold aggregates.

In summary, both experimental results on gold colloids as obtained from light scattering and microscope imaging measurements prompt us to believe that optical potential due to non-uniform electric field distribution around the aggregates may play an important role in determining the effective interaction between gold colloidal particles in the sol. Our experiments and simulation studies indicate that more detailed investigation is needed on the aggregation of gold colloidal particles of different shape and sizes. This can also help in the refining the theoretical models in order to achieve a more accurate scenario of their fractal behavior. Based on our discussion on Fig. 2 and Fig. 3 we are of the opinion that the effect of light needs to be considered in order to understand the detailed morphology and characteristics of such self-assembled gold aggregates.

## Experimental Method

Gold colloid is prepared following the well established method of citrate reduction [31]. 240 mg of HAuCl_4_ is dissolved in 500 ml of water (1.4 mM) and heated under vigorous stirring. A solution of 1% sodium citrate (50 mL) is added. The boiling is continued for 1/2 hour. Sodium citrate acts both as reducing agent and stabilizing agent. The pH of the solution is 6. Assuming that all the HAuCl_4_ is converted to a dispersion with diameter 22 nm, the number of particles in the solution is 10^15^/cm^3^. The measured value of conductivity of the water used is ~3.5 μS/m at 25°C.

No other chemical is added to the sol to induce aggregation. The aggregation of the particles, discussed in this article, is believed to be diffusion limited in the sol. The particles undergo random walk in the sol. When the separation between two particles is below a certain critical value, they pull each other.

Particles in the sol are characterized by measuring their surface plasmon resonance bands using a UV-1800 UV−vis spectrometer (Shimadzu, North America) for the spectral range between 400 and 700 nm.

Fractal behavior of colloidal aggregates in the sol was verified by static light scattering measurements. The as-prepared gold sol was diluted by 1:2, 1:4, 1:8 and 1:10 volume ratios to avoid the effect of multiple scattering of light. For each sample, data are collected at scattering angle between 0° and 15° with approximately uniform spacing. The excitation sources are the 633 nm and 543 nm lines of He-Ne laser. The intensity of the incident light was kept ~2 mW. The scattered light intensity is measured by a Si photodetector. To study the effect of resonance wavelength, similar light scattering measurements were carried out with 1:8 diluted sol using 488, 532, 632 and 785 nm lasers as excitation source.

To observe fractal morphology, the as-prepared sol was diluted to 1:8 volume ratio. To record the microscope images of the aggregate, drops of 5 μl each of this diluted sol was transferred on a thoroughly cleaned glass plate. Then they were allowed to dry in two different environmental conditions, in light and in darkness. Temperature was kept same in both cases. The radius of evaporated drop was ~2 mm. The images of the aggregates were studied under an optical microscope (BX 41, Olympus, Japan) using ×10 objective lens.

To probe the effect of substrate on fractal formation, we modified the glass-surface by changing the density of free reactive SiOH groups on it. This was accomplished by treating the glass with alkali hydroxides, particularly by sodium hydroxide (NaOH)^32^,^33^. We have dipped the glass substrate in NaOH solution of different concentrations and for various durations. All experimental results reported in this article were repeated several times on freshly prepared colloidal sol to confirm their reproducibility.

## Author Contributions

S.Bh. and A.R. were involved in experimental part of the work. S.N. and S.Ba. carried out the simulation. All authors reviewed the write-up.

## Supplementary Material

Supplementary Information Supplementary information
