# Supplementary material for: Does Shining Light on Gold Colloids Influence Aggregation?
Source: Sci Rep. 2014 Jun 9;4:5213. doi: 10.1038/srep05213 (PMC4048914; doi:10.1038/srep05213)
Supplement: Supplementary Information [file srep05213-s1.doc]

**Supplementary Information**

**Does Shining Light on Gold Colloids Influence their Aggregation?**

Susmita Bhattacharya^1^, Suda Narasimha^2^, Anushree Roy^1^*, Soumitro Banerjee^3^

^1^Department of Physics, Indian Institute of Technology Kharagpur, India

^2^Department of Physical Sciences, Indian Institute of Science Education and Research, Kolkata, Mohanpur Campus, India

^3^Department of Physical Sciences, Indian Institute of Science Education and Research, Kolkata, Mohanpur Campus, India, and King Abdulaziz University, Jeddah, Saudi Arabia

**S1.**

Figure S1. Power spectra for the images in Fig. 2(b) and (c). To estimate the slope, the range of chosen *k* corresponds to the length (*l*) of the fractal structure (*k=2π/l*).

**S2. BD simulation has been carried out using DLVO potential**

U(r) = U_R_(r)+U_A_(r), ………………………………………………………………........(1)

The Coulomb potential is taken as $U_{R}(r)=2\pi\varepsilon_{0}\varepsilon_{r}a\Psi^{2}\ln[1+\exp(-\kappa s)]$ for *κa*>>1,[[1]](#endnote-2)

where,  *s=r-2a* is the surface-to-surface separation between gold particles. *a* is the radius of a single gold particle. *є_r_,* and *є_0_* are the relative dielectric constant of the medium and the permittivity of free space, respectively. Ψ is the particle surface potential, and *κ* is the inverse Debye screening length. In our simulation, the required parameter values are set as follows: *a*=11 nm, *є_r_*=80, T=300 K, Ψ=−38 mV (measured ξ-potential for the as-prepared as well as diluted colloid), *є_0_* = 8.85×10^-12^ m^-3^ kg^-1^ s^4^ A^2^. The value of *κ* has been estimated to be 5.6×10^7^ m^-1^ following the relation mentioned in ref [[[2]](#endnote-3)], $\kappa=(2e^{2}cN_{A}/\varepsilon_{0}\varepsilon_{r}k_{B}T)$. *c* is the concentration of electrolyte (here *c*=0.3), *e* is the electronic charge.

In the van der Waals interaction $U_{A}(r)=-\frac{H}{12}\left[\frac{1}{x^{2}-1}+\frac{1}{x^{2}}+2\ln\left(1-\frac{1}{x^{2}}\right)\right],$ *H* is the effective Hamaker constant (75.5 *k_B_T).*  *x=r/d* and *r* is the centre to centre distance between particles.

In the simulation the resultant force acting on the each colloidal particle follows the relation $F=-\frac{d^{2}U(r)}{dr^{2}}+W,$ where *W* is the random force acting on the each colloidal particle due to solvent molecules in the sol. The simulation time step was set in the range of 10−100 µs. We enforced periodic boundary conditions to minimize wall effects. From the volume fraction and density relation the number of monomers has been calculated for a given volume fraction or areal fraction. If the value of *s* is greater than *1.5a*, the interaction potentials is taken as zero. To investigate *D_f_* of the aggregates for different volume fractions of the colloidal particles in the sol, the ratio of the number of monomer and solvent molecules was varied.

For nanoparticles stabilized by long chain ligand molecules, repulsive interaction due to free energy of mixing of ligand molecules is considered in a few recent reports[[3]](#endnote-4),[[4]](#endnote-5) , instead of the electrostatic repulsion term in the DLVO potential. In our case, the as-prepared particles are mainly stabilized by surface charge. Moreover, as citrate anions are very small in size (the maximum length of an ion attached to the metal surface is estimated to be 0.717 nm)[[5]](#endnote-6), they cannot be treated as long chain polymers. Thus, our simulation is based on electrostatic repulsive part only, neglecting free energy mixing of ligand molecules.

1. Verwey, E. J. W. & Overbeek, J. Th. G., Theory of the Stability of Lyophobic Colloids, (Elsevier, Amsterdam, 1948). [↑](#endnote-ref-2)
2. Ansell, G. C. & [Dickinson](http://publish.aps.org/search/field/author/Eric Dickinson), E. Short-range structure of simulated colloidal aggregates. *Phys. Rev. A* **35**, 2349–2352 (1987) [↑](#endnote-ref-3)
3. Khan, S. J., [Pierce](http://pubs.acs.org/action/doSearch?action=search&author=Pierce%2C+F&qsSearchArea=author), F.,  [Sorensen](http://pubs.acs.org/action/doSearch?action=search&author=Sorensen%2C+C+M&qsSearchArea=author), C. M. &  [Chakrabarti](http://pubs.acs.org/action/doSearch?action=search&author=Chakrabarti%2C+A&qsSearchArea=author), A. Self-assembly of ligated gold nanoparticles: phenomenological modeling and computer simulations. *Langmuir* **25**, 13861–13868 (2009). [↑](#endnote-ref-4)
4. Khan, S. J., Sorensen, C. M. & Chakrabarti, A. Computer simulations of nucleation of nanoparticle superclusters from solution. *Langmuir*  **28,** 5570–5579 (2012). [↑](#endnote-ref-5)
5. Huang, T., Nallathamby, P.D. & Xu, X-H. N. Photostable single-molecule nanoparticle optical biosensors for real-time sensing of single cytokine molecules and their binding reactions. *J. Am. Chem. Soc*. **130**, 17095–17105 (2008). [↑](#endnote-ref-6)
